# On Carbonagogue Medicines

**Published:** 1851-06

**Authors:** Lewis Rogers

**Affiliations:** Professor of Materia Medica and Therapeutics, in the Medical Department of the University of Louisville


### WESTERN JOURNAL

OF

### MEDICINE AND SURGERY.

JUNE, 1851.

### Art. I.—*On Carbonagogue Medication.* By Lewis Rogers, M. D., Professor of Materia Medica and Therapeutics, in the Medical Department of the University of Louisville.

Animal Chemistry has been diligently engaged, for some years, in exploring the composition and structure of the fluids and solids of the human body, and has developed a variety of truths both novel and interesting to the physiologist. Indeed, so much has been achieved by the varied and searching investigations of the chemist and microscopist, that the physiology of the present day has passed through a revolution so complete, as scarcely to be recognised now as the branch of science passing under that name twenty years ago.

The revolution in physiological doctrines, and the more accurate and refined conceptions now entertained of the structure and functions of the human system when in health, have, to a marked extent, modified the doctrines of pathology, and while expanding, have improved our knowledge of the phenomena and laws of morbid processes. The crude theories of humoralism and solidism, once so prevalent and so confidently announced, have been succeeded by the development of a great mass of both independent and connected facts, not yet sufficient, it is true, to lead to conclusive and unexceptionable generalizations, but, as the product of cautious and practised inquiry, destined to furnish the materials from which more perfect general principles will be educed, at a period not very remote. At no time in the history of medicine has research been so industriously and accurately prosecuted, and the laborers in the various departments of medical science so richly rewarded by valuable and reliable results.

Correct physiology and correct pathology, form the basis of sound and philosophical therapeutics; without such a basis the practice of medicine is little better than mere empiricism, established upon a vague and uncertain experience, and groping its way, without the guide of laws or principles, to doubtful and often disastrous results.

While physiology and pathology have been advancing with so much rapidity, it is not a little remarkable that therapeutics, to subserve the purposes of which these are alone valuable, except as matters of abstract science, have advanced in a degree by no means commensurate. "While animal chemists and physiologists are daily pointing out the composition of the solids and fluids of the animal economy, it is a matter of great surprise that we so rarely hear of pathologists applying with a view to the lessening disease, the doctrines which the former have established and promulgated." Whilst the physiologist and pathologist of the present day enter very profoundly into the arcana of the special subjects of their investigations, and with the microscope and the implements of chemistry follow up the matters involved in their inquiries to the utmost limits of analysis and synthesis, the therapeutist is content, for the most part, to deal in indefinite generalities, and to address his remedies to broad and comprehensive indications, the fulfilment of which, in the summary manner usually adopted, will often be found, upon a rigid analysis of the component elements of these indications, to include a contradictory course of medication, one part of which may entirely annul another. The therapeutic application of modern physiological and pathological discovery has, to be sure, been successfully essayed in a few specialties, but as a general truth, it may be confidently asserted, that the greater part of the so-called therapeutic principles are the precepts of a random experience, and not educed from a philosophical consideration of healthy and morbid processes, and the most direct methods of subjecting the latter to the control of the former.

In the treatment of the various forms of fever, and indeed in the treatment of most diseases, both acute and chronic, a leading and controling indication is the restoration of the secretions; such is the common phraseology of most practitioners of medicine, and remedies are variously and vigorously addressed, with the view of attaining this prominent purpose. Now, what is expressed and generally understood by a restoration of the secretions in fever? The skin is hot and unperspirable; the kidneys excrete a diminished amount of urine; the liver, and the glandular and exhalent structures involved in the mucous membrane of the alimentary canal are slow in the effusion of their appropriate fluids; in a word, there is a diminution or arrest of the normal functions of the organs concerned in the important offices of secretion and excretion, and particularly of the latter. This arrest of the healthy excretory processes, while it is probably one of the earliest of the noxious effects of the peculiar poison engendering the fever, becomes often in the progress of the affection, a leading source of difficulty and danger, by inclosing in the system excrementitious elements, the steady and unfailing elimination of which is now deemed essential to the health of the economy. The general purpose held in view by practitioners in their efforts to restore the secretions is to induce a soft and moist state of the skin, the discharge of a natural or an increased amount of urine, an open state of the bowels, and free bilious evacuations. When the treatment of a case of fever is so managed as to realize these important effects, it is generally successful, and the great end of therapeutics is achieved.

A few years ago, before animal chemistry had occupied the attention of the profession to any great extent, and before the wonderful actions constantly going on in the laboratory of the human body were in some measure revealed, very limited and imperfect ideas were entertained in reference to the important chemical as well as vital offices performed by the several secretions and excretions above enumerated. By the lights of animal chemistry, physicians of the present day are enabled to discern more fully the real purposes of these processes. As regards the skin, a restoration of cutaneous transpiration is now understood to include, not only the effusion of a certain quantity of water, the equalization of a lost balance of the circulation, and a regulation of the animal temperature, but also to involve the very essential elimination of a considerable amount of carbonic acid, of effete nitrogenous principles, and of a variety of organic and inorganic salts, the retention of which is known to be always baneful, and in some inferior animals has been proved by experiment to be fatal in its effects. As regards the restoration of the urinary excretion, the physiological or chemical therapeutics of the present day looks beyond the mere evacuation of a certain number of ounces of an amber-colored fluid, and recognises the important office of this excretion to be, the deportation from the system of worn-out carbonaceous and nitrogenous elements, the product of secondary assimilation, or returned to the circulation as mal-assimilated or surplus food not appropriated by primary assimilation. According to chemical therapeutics, the restoration of the urinary excretion implies the elimination from the system of water holding in solution the materials above designated, with a variety of salts, the accumulation of which in undue quantity is productive of the most dangerous effects.

While it is the office of the kidneys largely to afford escape to the refuse nitrogen of the system, it is the province of the liver to secure an outlet for the waste carbonaceous matters circulating in the bloodvessels, and deposited there as the product of the metamorphosis of the exhausted tissues, or thrown back as an unappropriated portion of a redundant alimentation. The liver, aided by the lungs, largely depurates the system of its superfluous hydro-carbon; when the one organ is faulty in its action, the other may, to a limited extent, act a supplementary part, and when both fail, disorder and disease are very certainly produced by the presence of effete principles thus denied a ready evacuation. There is no secretion so sedulously watched in the treatment of all forms of disease as this of the liver; its restoration when absent or deficient, its correction when vitiated, its free flow in all stages and conditions of general and local disturbance, are esteemed of more essential importance than all of the other secretions and excretions combined. Popular as well as professional observation attests the correctness of this opinion in a very large number of both acute and chronic affections, yet when the subject is scrutinized by the mind of the chemical therapeutist, and the view is carried beyond the indefinite and summary estimate generally formed of the fluid called bile, to a chemical analysis of its elements, and the very probable purposes subserved by the excretion of the compound formed of these elements, it can be readily conceived that cases may frequently present themselves in which it may be necessary to retard this function, with the design of preventing a too rapid waste of the elements which it separates. Some illustrations of this necessity will be adverted to in a subsequent part of this paper. Of the entire quantity of bile formed by the liver, a very small proportion only is evacuated by the bowels in healthy states of the system, the greater part being absorbed after it reaches the intestinal canal, and excreted by the lungs in the form of carbonic acid gas. To estimate properly then the benefits to be derived, or the results to flow from a restoration or increase of biliary secretion, it is requisite to be guided somewhat by the lights of animal chemistry, to bear in mind the intimate composition of this fluid, and its probable physiological purposes, and with these, to connect the pathological condition of the fluids and solids, in the case under treatment. It may be that the system is surcharged with carbonaceous matters, or on the other hand, it may be the fact that these have been already unduly wasted, and that a restoration of carbon is far more essential to the cure of the patient, than the restoration of biliary excretion, and a further loss of this essential element of vitalised structures. Whilst the prevalent therapeutics are disposed to prescribe an invariable stimulation of this function, therapeutics, based upon animal chemistry, suggest a cautious discrimination, and resisting the routine adherence to an antiquated practice, regard the hepatic secretion as a composite fluid formed with some definite design, and not as a vague and meaningless production.

Physiological and chemical inquiry informs us that a certain number of gaseous and solid bodies constitute the materials of which the healthy body is composed; these gaseous and solid matters, under the influence of vital action, are arranged in a definite and particular manner, and hold to each other certain fixed and determinate relations, the unbroken preservation of which is essential to the sanity of the organism. The solids and fluids of the body present a uniformity of composition from which there can be only a very slight departure consistently with health; any material disarrangement, increase, or decrease of the gaseous and solid elements from which they are so curiously and wonderfully formed, must necessarily vitiate their original purity, and develop those disturbances of structure and function which constitute disease.

Of the gaseous and solid substances which enter into the composition of all animal structures, none exist in greater abundance, or perform a more important part, than carbon and its compounds. In one form or another, carbon enters into the composition of most of the animal tissues, and constitutes a large element in the various products of destructive metamorphosis of these tissues. In this way, through the medium of the nutritive fluid circulating through every part of the system, this substance is constantly received by, and as constantly expelled from, the body. Its regular reception and its regular expulsion are alike necessary to the healthful processes of the economy. Its undue reception, its too profuse elimination, and its preternatural accumulation are equally obstructive of these processes. The accumulation of carbon in the system, either as the result of too large a supply, or of a tardy action on the part of its proper organs of excretion, does not seem to have had that due weight given to it, in the causation of disease, to which it is probably entitled. Dr. Carpenter, in speaking of those ingredients of the tissues which are no longer in a state of combination, that fits them for their offices in the animal economy, remarks: "To separate these ingredients from the general current of the circulation, and to carry them out of the system, is the great object of the excretory organs; and it is very evident that the importance of the respective functions of these will vary with the amount of the ingredient which they have to separate, and with the deleterious influence which its retention would exert on the welfare of the system at large. Of all these injurious ingredients, carbonic acid is, without doubt, the one most abundantly introduced into the nutritive fluid; and it is also most deleterious in its effects on the system, if allowed to accumulate."

Toxicologists differ somewhat in opinion as to the precise mode in which carbon exercises its hurtful influences when applied to the human body in the form of carbonic acid gas. Some think that it is altogether negative in its action, producing asphyxia by excluding atmospheric air. Christison and other accurate observers have, however, satisfied themselves, that this gas is endowed with powers positively and essentially poisonous. An atmosphere impregnated with five, or even three per cent of this gas will occasion unpleasant and sometimes fatal effects in a short time. It acts upon the nervous system with its greatest energy and promptitude, through the medium of the lungs, but its influence is not restricted to the pulmonary surface; it will be felt, very speedily and very sensibly, when brought in contact with the mucous membrane of the stomach; and when the external surface of the body is immersed in it for a short time, its characteristic poisonous effects are certainly induced, even though pure atmospheric air be admitted freely to the lungs during such immersion. Other gaseous compounds of carbon, as carbonic oxide, and carburetted hydogen, are also poisonous, though probably not to the same degree. The carbon is no doubt the essential basis of the poison in these several gases, and, as a question of inferential reasoning, it would seem to make very little difference, so far as its effects upon the powers of life are concerned, whether it be brought to bear upon the system through the lungs, stomach and skin, in a gaseous form, or accumulate gradually there, as a retained excretion, from a failure of the eliminatory organs, or a redundant supply of the materlals from which it is separated. Apart from all reasoning on the subject, there are several states of disease, such as jaundice and ischuria renalis, which are familiar and acknowledged illustrations of the noxous effects of the retention of matters in the system, one of the important elements of which is carbon.

A more correct appreciation of the results likely to flow from the retention of a poison like carbon, in undue quantity in the system, may be formed by a previous reference to the amount normally excreted by a person in health, every twenty-four hours. A robust, healthy adult is supposed, according to the best authorities, to excrete by the lungs, in twenty-four hours, about 3840 grains, or 8 ounces troy, of carbon. The kidneys excrete in the same time about 433 grains of urea, 13 grains of uric acid, and from 50 to 60 grains of coloring matter, all of which will yield, upon analysis, near 120 grains of carbon. The skin excretes about 107 grains of organic matter, according to Seguin, mostly nitrogenous, but containing carbonaceous compounds sufficient in amount to yield 35 grains of carbon. The exact quantity of bile secreted by the liver, in the period of a day, has not been accurately ascertained; some ten or twelve ounces are discharged into the duodenum, but a large and the most essential part of this, the biline, is again absorbed, and carried into the circulation, to subserve other purposes, and probably, by the union of its elements, carbon and hydrogen, with oxygen, to assist in the maintenance of animal heat; a considerable amount of it no doubt escapes by the lungs in the form of carbonic acid and water. It is a well ascertained fact, that a very small proportion of the bile daily elaborated is contained in the feces passed in a state of health; estimating this amount at about 1 ounce, and we shall have something near 342 grains of carbon evacuated by this channel, in the twenty-four hours. Adding together the quantities thus arrived at, a total of more than 9 ounces of carbon is ascertained to be daily thrown out of the body, by the several most active emunctories. The estimates here given do not correspond exactly with those of Liebig and others, but for the purpose of illustration, they are sufficiently accurate. This carbon has performed its offices in the economy—it is now refuse matter, the product of textural decomposition, and demands a prompt egress from the system—retained, in a simple or compound form, and in progressively accumulating quantities, it becomes endowed with the powers of a poison—pent up in the blood to the extent of one-half, or even one-fourth of the quantity which we have estimated to be daily eliminated, it cannot fail to engender dangerous and fatal disease.

The lungs being, as we have seen, the channels through which much the larger part of the waste carbon of the system is eliminated, it becomes a matter of interest to know, to what extent disease in these structures, and in other parts of the body, increases or lessens the amount thus disposed of. Minute and systematic observation on this point is yet required; the few facts which have been ascertained have only been noted as of physiological interest, no practical application having been made of them, either to pathology or therapeutics.

In the acute eruptive diseases, it has been found that an increased amount of carbonic acid gas is exhaled by the lungs. This fact is readily explicable. In these diseases, there exists an unusual tendency to decomposition in the fluids and solids; metamorphosis of the tissues takes place with uncommon rapidity, and the blood is replete with the disintegrated matters thus forced upon it; the dark color of this fluid, often remarked in such affections, indicates its surcharged condition. The lungs afford the most convenient and efficient outlet through which a depuration of these products may be effected. In addition to this, one of the offices of the skin is to eliminate carbonic acid; when this tissue becomes the subject of disease, this office must be abridged, to some extent, and an increase of excretory action be thus imposed on other parts. The nervous and vascular excitement necessarily attendant upon this sudden increase of functional activity in the lungs must predispose to, if it do not actually develop morbid action in these organs. In this way, without reference to the peculiar affinity of the specific poison engendering the exanthem, may we account, in some measure, for the well-known proneness to pulmonary complications characterizing some of the diseases of this class.

Cold weather and cold climates cause the exhalation of an increased amount of carbonic acid by the lungs; this results from the increased consumption of the carbonaceous elements of the body necessary to the generation of the requisite amount of animal heat. This increased amount of carbonic acid is mainly extricated through the medium of the respiratory organs, the functional activity of which must be correspondingly increased. In this way, may we partially account for the great preponderance of pulmonary affections in such seasons and such climates. Warm weather and warm climates diminish the functions of the lungs, relieve the necessity for the great elaboration of animal heat, lessen the amount of carbonic acid evolved by these organs, and impose upon the skin and liver the office of expelling the effete carbon of the body. In this way a very rational explanation is afforded of the various cutaneous and hepatic diseases incident to warm climates and warm seasons of the year.

From our knowledge of the additional functional labor imposed upon the respiratory organs, during the progress of the acute exanthemata, a practical precept of much value in the treatment of these affections may be educed. When a predisposition to pulmonary disease exists, or when acute pulmonary inflammation has been developed, in the course of these affections, the lungs should be relieved, as far as possible, of the onus of eliminating the superabundant carbon, by carbonagogue medication addressed to the liver, to the kidneys, and to othor organs that may be forced into the performance of a part of this function. By the vital and *chemical* derivation thus induced, the lungs may be protected, in some degree, from the danger of over-excitement, and the general system at the same time relieved of a deleterious agent. The restoration of the secretions, by medicines severally having an affinity for them, is but another name for the practical principle here indicated.

In chronic diseases of the skin involving a considerable extent of surface, observation has detected the presence of an increased quantity of carbonic acid, in the pulmonary exhalations. By the consequent exaltation of functional activity, irritation, chronic inflammation, or some other form of organic difficulty may be engendered in the lungs; or if disease, or predisposition to disease already exist in these structures, they may fail to perform their supplementary office with success, and if other organs capable of eliminating carbon, do not spontaneously come to the rescue, or be not solicited to do so by appropriate medicines, the system must suffer from the presence of effete and noxious elements. Hence, the utility in such cases of the occasional, and even persistent resort to carbonagogue medication, in some one or more of its forms. On the other hand, it is a generally recognised doctrine, that many cases of chronic skin disease are due primarily to the presence in the blood of excrementitious matters, which have been detained there by a failure of some of the excreting organs. This functional failure may be resident in the lungs, in the liver, or in the kidneys, more frequently in the two latter than in the former. This excrementitious matter is usually composed of organic as well as inorganic elements, and carbon is very sure to exist as an abundant constituent. External treatment by lotions, baths, ointments, and other similar superficial means is usually impotent in affording relief; the only successful method of cure consists in that which awakens, and persistently maintains, more free action in the great purificative organs. Hence, the well known efficacy, in such forms of disease, of expectorants, diuretics and cholagogues, which in truth relieve, in a great measure, by their power as carbonagogues. What is the real and ultimate curative influence of an expectorant? Its obvious and appreciable and commonly recognised effect is the removal from the surface of the bronchial tubes of various fluids which coat that surface, and obstruct the free ingress of atmospheric air; by promoting the excretion and expulsion of these fluids, the oxygen of the atmosphere readily gains access to the minute vessels of the lungs, combines with the blood, pervades the body, forms carbonic acid with the effete carbon of the system, returns to the lungs, and is discharged through the avenues of its entrance, having effected that important process denominated decarbonization of the blood. We resort to expectorants then, not simply to disgorge the vessels and tubes of the lungs of certain fluids that may be lodged there, but for the higher end, of more thorough aeration and decarbonization of the blood. Under this chemical view of the mode of action of expectorants, they may with great propriety be ranked as carbonagogue remedies. We give diuretics and cholagogues upon similar principles; not simply to evacuate an increased amount of urine and bile, but with the more radical view of unloading the system of the chemical elements of which these fluids are composed.

In chlorosis and amenorrhea, and during the existence of pregnancy, there is also a well-marked increase in the amount of carbonic acid exhaled by the lungs. From the period of puberty, when menstruation begins, up to the time of the cessation of this function, not more than the normal amount of this gas is eliminated with the expired air, the quantity permanently increasing upon the final arrest of the catamenial flux, and undergoing temporary exaltation whenever any accidental interruption to this function is experienced. These facts would seem to attach something of a depurative character to the monthly evacuation of dark-colored sanguineous fluid from the uterus, and to afford foundation for the idea so prevalent in the popular mind, that this organ relieves the system, from month to month, of impure and redundant principles, the accumulation and retention of which are charged with the causation of countless ills. The idea that the uterus is concerned in the elimination of useless carbon from the system is sustained by other considerations than those connected with the increased amount of carbonic acid thrown off by the lungs, under temporary or permanent suspension of its normal duties. Dr. Butler Lane, in a paper "on Functional Diseases of the Liver associated with Uterine Derangement," advances, and very ingeniously sustains, an opinion of this kind. Dr. Lane, making use of the tables, constructed by Dr. John Reid, of the relative weights of organs, ascertains the important anatomical and physiological facts, that the average weight of the liver in the female is much less than in the male, and that this diminution of the weight of the female liver is particularly marked at the period of menstruating and childbearing, from twenty to thirty years. Availing himself of the analysis made by Dr. Simon of the portal and hepatic venous blood, as well as of the menstrual fluid, he "conceives an affinity to subsist between the last, and the portal blood, and therefore the probability of an hepatico-uterine consentaneity." In support of this opinion, he refers to the very common occurrence of alvine derangement during the menstrual period, and expresses his belief that "at the approach of the uterine excitement, the function of the liver is performed imperfectly; the arterial blood becomes to some extent impure; that this very impurity is the essence of the peculiar constitutional state that exists; and that it is by means of the uterine secretion the blood undergoes the necessary purification by which the system becomes relieved." He states that there may be an association or antagonism of morbid action between the two organs, in their various disorders, but "it rarely happens that the natural secretions of the liver and uterus are consentaneously in excess, and when such is the case, it chiefly originates in the course of other diseases, and not unfrequently is the result of undue medical influence." According to these views of Dr. Lane, the uterus, besides performing its part in the great office of reproduction, is economically designed by nature to assist in expurgating the female system of refuse carbonaceous matter, a task more exclusively performed by the liver, in the male. This chemical mode of considering the final though partial office of the uterus, when therapeutically applied to failures and vitiations of its function, compels us to rank the class of medicines heretofore denominated emmenagogues, in the same category with cholagogues and expectorants, for they are in truth carbonagogue in their action, if we consider only the composition of the fluid eliminated, without reference to the organ or the monthly periods, through which, and at which the important elimination takes place. This method of considering such medicines may not extend our knowledge of the precise mode of accomplishing their purpose, nor enable us to administer them with more certainty of effect, yet such a classification greatly simplifies this difficult subject, and brings therapeutics up to the level upon which organic chemistry has placed physiology and pathology. If the great and ultimate purpose of cholitic, expectorant, and emmenagogue medication be, to promote the full and normal decarbonization of the blood, scientific accuracy, if not didactic convenience, will be consulted, by arranging all articles of the materia medica capable of effecting this end, under the one comprehensive class of carbonagogues.

Connected with the vicarious duties which the lungs assume in cases of chlorosis, amenorrhea, and pregnancy, as efferent organs of carbonaceous matter, some useful therapeutic hints suggest themselves, pertaining to the remedial management of such cases. If acute or chronic organic disease exist in the lungs associated with a cessation of function on the part of the uterus, a state of case which often occurs, the pulmonary exhalation of carbonic acid may not be sufficiently augmented to unload the system to the requisite extent. Experimental inquiry has ascertained that when the lungs are suffering from somewhat extensive organic disease, a diminished exhalation of carbonic acid is the consequence. Under such complicated conditions of deranged action, it will become necessary to the relief of the system to address carbonagogue medicines, from time to time, to the other organs capable of performing this species of depuration. Hence, the utility of the occasional administration of the so-called cholagogues, diuretics, and diaphoretics, the benefit of which experience attests. The kidneys, though mainly depurating the blood of its superfluous and waste nitrogenous elements, perform the same office, to a limited extent, as regards the carbonaceous products. Similar remarks are applicable to the skin. By stimulating the functions of this tissue, by frictions, bathing, and medicinal diaphoretics, increased facility will be afforded to the egress of its gaseous and fluid exhalations. Among these, carbonic acid is constantly present. In this sense, diaphoretics and other remedial means calculated to exalt the functions of the skin must be considered carbonagogue agents. The skin in a state of health has been proven to eliminate a certain quantity of carbon, other organs performing, at the same time, their share of the same office; there is sufficient reason to assume, that, when these latter are crippled in their power, the skin is every way competent to take on an increased and substitutive form of action.

Amenorrhea is a pathological effect dependent upon a variety of causes. It may be associated with a hyperemic or an anemic condition of the general habit exclusively, or one or the other of these constitutional conditions may exist in connection with disease in the uterus and its appendages. A diversity of distressing symptoms, and ultimately of distant complications co-exist with it, products of the original general or local lesion, or dependent upon the suspended function more immediately, and due to a retention of excrementitious principles that hitherto have received a monthly emission from the system. This latter humoral view of the case is the one invariably taken by the patient. She may be the subject of incurable disease in the lungs, in the kidneys, in the heart, or in the liver, and such disease may, beyond all question, have induced secondary lesions in the functions of the sexual system, yet no argument can convince her that the first link in the chain of symptoms does not connect with the uterus. Whilst the intelligent physician will firmly rely on the accuracy of his diagnosis, and direct his treatment in conformity with the initial or radical indications of cure, some regard is justly due to the malign influences which may be exerted by the retention of effete and excrementitious materials. Nature herself often testifies to the value of this intercurrent medication by the spontaneous development of vicarious hemorrhage from the nose, lungs, stomach, and bowels, and even from more superficial parts of the body; and this, too, in cases complicated with the most serious and extensive organic lesions in vital parts. By the timely administration of carbonagogue medicines capable of acting through other and unobstructed outlets of the system, these spontaneous but alarming eruptions may possibly be averted, and the comfort of the patient materially enhanced. An open state of the skin, of the kidneys, and of the liver, with a free access of oxygen secured to the lungs by pure air and exercise, will largely conduce to the ease if not to the cure of such cases.

According to Dr. Lane, menstrual derangement strongly inclines to hepatic disturbances; a reference to statistics establishes the greater proneness of females than males to biliary disease. The same is to some extent true in regard to the respiratory organs. Protracted amenorrhea, dysmenorrhea, and chlorosis are very sure to awaken pulmonary difficulty, particularly if there exist any hereditary proclivity to such affections. Hence the necessity in the treatment of such cases of distributing the efforts of vicarious decarbonization, by medication variously directed to the several organs competent to the service, and of not imposing upon any one organ too heavy a burden.

Pregnancy, or the arrest of menstruation incident to this condition, developes an increased exhalation of carbonic acid by the lungs. Upon the principle that an increase of functional activity generates an increased liability to disease, females during the period of utero-gestation should be more prone to affections of the pulmonary organs. We have not data sufficient to justify a definite judgment on this point, in reference to a large part of the acute and chronic affections of these organs, but as regards tubercular phthisis, the sentiment of the profession is decidedly adverse to such a conclusion. Pregnancy has long been considered to avert the development and to mitigate the progress of tubercular consumption of the lungs. My own observations, confirmed by the more extended experience of others, induce a doubt of the correctness of the prevalent opinion. Dr. Grisolle, in a paper read before the medical section of the Academy of Medicine of Paris, on the "Influence which Pregnancy and Phthisis Exercise Reciprocally Upon One Another," assumes the very opposite ground. Facts which he has observed induce the following conclusions: that conception is a rare occurrence during the existence of confirmed phthisis; that the first symptoms of tubercular deposits become very often suddenly evident in the course of pregnancy, particularly in the three or four first months; that pregnancy has been repeatedly observed to act as an exciting cause in women predisposed to the organic disease; that the pulmonary affection went through its stages more rapidly with pregnant women than with unimpregnated subjects; and, lastly, that the organic changes appeared to be stayed immediately after delivery had taken place, provided the patient had not been in a very advanced stage of phthisis.

Assuming an increased liability to phthisis during the existence of pregnancy, as a pathological fact sustained alike by speculative considerations and clinical observation, prophylactic measures based upon the special etiology above suggested might have some useful preventive influence upon females known to be tainted with a tubercular predisposition. Vital as well as chemical derangement unquestionably plays an important part in the production of the tubercular diathesis and in the deposition of tubercles; the former, however, is far more inscrutable in its mode of action and less directly amenable to the influence of remedies.

In acute and chronic organic disease of the lungs, the quantity of carbon eliminated by this channel is sensibly diminished, and this notwithstanding the efforts for relief made by the increased frequency of the respiratory movements. The lungs are designed to afford an interchange of communication between the atmosphere and the blood, by which oxygen is imparted to the latter and carbon to the former. This interchange will be interrupted just in proportion as organic lesion obstructs the ingress of atmospheric air to the extensive recesses of the pulmonary tissue. In acute diseases, the interruption is sudden and violent, gives rise to tumultuous disturbance, and leads to prompt issues. This tumultuous disturbance is largely due to the sudden arrest of sanguineous decarbonization, and is productive of suffering and danger in a degree commensurate with the intensity and duration of this arrest. The case is one of acute poisoning from the occlusion of oxygen and the inclusion of carbon. The various symptoms of local and remote suffering attendant upon acute pulmonary disease arise from topical and general disturbance of the nervous and vascular systems, but with the whole is blended this one dominant and prevailing element, insufficient oxygenation and decarbonization of the blood.

In chronic affections of these organs, which are slowly developed, such as do not seize rudely or violently upon a large extent of breathing surface at once, a train of symptoms, similar in kind but differing in intensity, is developed, and similar ultimate effects are slowly though no less certainly induced. The patient suffers and dies from a deficiency of oxygen and an excess of carbon. The disease may be pleurisy, or pneumonia, or bronchitis, or it may be chronic empyema, or tuberculization, and the signs and symptoms may differ, and the sympathetic phenomena be infinitely diversified, yet, when they are all analyzed and reduced to their simple elements, with the view of ascertaining in what way they really assail vitality, the humble and familiar chemical conditions, so frequently adverted to, assert their power and are unquestionably entitled to the claim.

The structural lesions in these affections are hurtful only so far as they seriously interfere with the great and indispensable offices of respiration, the oxygenation and decarbonization of the blood. By an obstruction of this process, a poison is retained in the system, by which not only the part primarily involved is injured, but the vitality of every other part either diminished or annulled. Disease may extend from part to part, and from organ to organ, until the entire body seems to be involved, and it is the fashion to explain this progressive invasion of part after part by the vague term of sympathy, but rigid scrutiny will discover that imperfect decarbonization enters as a modifying if not a controlling element of causation.

In the treatment of acute inflammatory affections of the lungs, blood-letting and other antiphlogistic remedies are resorted to with the view of at once arresting inflammatory processes subversive of the functional and structural perfection of the organs. It is not necessary to the present purpose to enter into a consideration of the mode of action of the remedies most successful in the treatment of inflammation; it is sufficient to know that, whilst they achieve the important results aimed at, they are, at the same time, greatly subsidiary to the mitigation of the most dangerous pathological effect of the diseases under treatment. The lancet abstracts blood surcharged not only with fibrin but with other carburetted compounds; mercury possesses not only antiplastic and defibrinizing powers, but it is distinguished for its stimulant influence upon all the secretory and excretory organs, the skin, the kidneys, and very especially the liver, the great office of which is considered to be the depuration of the blood of its redundant carbon. The purification of the system through these outlets is a point in the treatment of such forms of disease second only to that which looks to the relief of the topical inflammation; if the remedies used for the latter did not happily combine modes of action competent to the former, it would become highly necessary to blend with those of a purely antiphlogistic character, such as are endowed with carbonagogue powers. Indeed, it is a question in the minds of therapeutists, whether the essential action of antiphlogistic remedies does not really consist in their power to hasten the processes of destructive assimilation, by which an increased quantity of disintegrated and exhausted matter is forced for elimination, upon the various excretory organs.

In the remedial management of chronic affections of the lungs, whether plastic or aplastic, considerations of the same kind are deserving of attention. Such affections injure and lead to fatal terminations, by inducing a slow asphyxia, with its attendant train of remote consequences, and the indications of cure call for the joint use of alteratives and entrophics, and those remedies calculated to unload the system of carburetted compounds, the presence of which constitutes the essential principle of asphyxia. By persistent attention to the condition of the skin, by such hygienic and medicinal means as will insure the preservation of healthy action in this extensive tissue, the system may be greatly disembarrassed and the progress of even incurable disease materially retarded. That the skin performs a respiratory function is made evident by the experiments of Fourcroy and Magendie, who found that animals become speedily and fatally asphyxiated, when the surface of their bodies was covered with an impermeable varnish, or enveloped in a caoutchouc dress. In the same manner, by the use of diuretics, and yet more by mercurial and other medicines which exert a decided cholagogue influence, palliation may be procured, when more radical relief is beyond the reach of art. Science here gives ready explanation to both popular and professional experience, for even patients soon learn the means by which they may secure for themselves at least temporary alleviation.

Between the lungs and the liver there exists a remarkable consentaneousness of function; this is true as regards both the physiology and pathology of the organs. This consentaneousness of action is evinced by phenomena which occur during the whole period of life, but is strikingly and particularly proven by the relative condition of the organs during fetal life, and immediately after respiration has been established. In the fetus, the liver is the decarbonizing organ, and the lungs are quiescent; accordingly, the former organ is very large and abundantly supplied with blood, while the latter are small and furnished only with a sufficient amount of blood for healthy nutrition. As soon as respiration commences, however, and the lungs assume the office of decarbonization, a prompt diminution in the size of the liver and in the quantity of blood circulating through it takes place, while a corresponding augmentation of the size and circulation of the lungs is at once obvious. This interchange of function between the liver and lungs, though more definitely marked in fetal life, is sustained, and displays itself, under special circumstances, in all subsequent periods. These physiological facts afford by analogy very strong confirmation of the opinion entertained by Dr. Butler Lane as to the similarity of function of the uterus and liver in the menstruating female, an opinion founded on the comparative weights of the male and female liver, and the quantity and quality of the blood in the portal, hepatic, and uterine vessels.

When the lungs are the seat of either acute or chronic disease, by which their function is abridged, the liver, when healthy, takes on increased activity, and a preternatural amount of bile is elaborated. This vicarious elimination of carbonaceous matter is of very constant occurrence. In some cases of great pulmonary embarrassment, as in extensive single or double pneumonia, or in pleurisy with copious effusion, bile is formed in quantities too profuse to find a ready egress through its ordinary channels; in cases of this kind, vomiting of bile may occur, or absorption and a jaundiced state of the entire body may ensue. Jaundice is a very common complication of pulmonary disease; vital chemistry explains, to some extent, the cause of its occurrence. When it complicates acute pleurisy or pneumonia, the compound affection is often denominated bilious pleurisy or bilious pneumonia, and is supposed to occur particularly in habits that have been subjected to the influences of marsh effluvia. It may occur, however, in habits that have never felt any such influences, solely as the result of the conservative physiological laws ever in operation, though it is more likely to arise when the system has been vitiated by the hypothetical cause alluded to.

Again, when the onus of eliminating carbon from the system is devolved upon the liver, by diseases impairing this function in the lungs, it may happen that this organ is not fully competent to the task. Under such circumstances, the vicarious duty must be performed by other more remote organs, and if not fully performed, carburetted compounds must accumulate in the system. Such accumulations do occasionally take place in the liver itself, as well as in other organs, giving rise to fatty degeneration of the parts involved. It is true that fatty degeneration is more an associate of the tubercular diathesis, than of tubercular deposits limited exclusively to the lungs, but in whatever connection occurring, it is an evidence of a defective excretion of the chemical elements of adeps. In default of a sufficient elimination of these principles through the emunctories especially designed for the purpose, an increased interchange may take place between the atmosphere and the blood, through the medium of the skin, and enough oxygen be introduced to effect the combustion of the retained hydro-carbon. In this way may be explained the persistent and obstinate paroxysms of febrile excitement so constant in some of these affections.

While pulmonary diseases impose upon other organs the duty of eliminating carbon from the system, and often occasion a permanent local or temporary general retention of it, the ultimate effect is to exhaust the tissues of the frame generally, and progressive emaciation is a prominent feature of some of these affections. The disturbed balance of excretory action, the persistent dislocation of physiological function engender, after a time, a morbid consumption of hydro-carbon and other elements of nutrition, and thus bring about the remarkable atrophy of the body. It is in this state of case, that carboniferous medicines, such as naptha and cod-liver oil act with such seeming excellency of effect; in many patients, for a time, arresting the emaciation and hectic fever, and in a few, according to authentic testimony, having even a more radically curative effect. The prevention of emaciation by these remedies is due to the supply of the chemical elements necessary to combustion, and to the formation of fat, while any radical benefit may be attributed to the free introduction of a healthier molecular base of the chyle, by which general nutrition is altered and improved, and plastic are substituted for aplastic deposits.

While imperfect decarbonization of the blood from pulmonary disease entails a large amount of labor and often disease upon the liver, the reverse of the proposition is equally true; protracted, or even temporary "torpor of the liver," or structural disease of any kind is very sure to awaken disorder of the respiratory system. While diseases, primarily seated in the lungs, arouse hepatic difficulty and its attendant train of dyspeptic symptoms, impaired digestion and biliary derangement are familiar and very constant precursors of pulmonary affections. The disturbances below the diaphragm are supposed to produce disturbances above it, through the agency of sympathy. A patient is said to have a cough or difficulty of breathing from sympathy with a disordered stomach or a deranged liver; such is the unsatisfactory explanation frequently given. The chemical physiology of the organs concerned offers, in part, a more satisfactory solution of this morbid association. A very common and troublesome symptom of hepatic disease, one exceedingly annoying to the patient, and often encountered by the practitioner, is the deep and prolonged inspiratory movement called sighing. This symptom is so marked and distressing in some cases as to approach the character of asthma, and doubtless, when it attains its maximum of intensity, it does constitute one of the forms of this affection. This sighing is not a mere emotional act; the deep inspirations are not mental in their origin; they are produced in obedience to the chemical wants of the blood, and are designed to convey an increased quantity of oxygen to that fluid, by which it may be relieved of the toxic influences of the carbon with which it is surcharged from diminished hepatic activity. This natural curative effort has been imitated by M. Tavignot, in the treatment of a nervous form of disease denominated by the French, migraine. This migraine is not a simple neuralgia of the head, but neuralgia "accompanied by a state of physical and moral prostration, during which the blackest ideas assail the patient. During an attack of this, which from former experience he was led to believe would continue twenty-four hours, M. Tavignot was induced by the hope that this condition of the nervous centres might result from a stasis of the blood in the sinuses of the brain, or from imperfect hematosis, to take several deep and rapid inspirations; and after a few efforts of this kind, he found himself completely relieved and able to resume his occupation. Other persons, similarly affected, have been in like manner relieved." Dyspeptic sighing is but one of a great number of illustrations that might be given, of what may be termed the chemical sympathy between the liver and the lungs; illustrations that simplify and solve the reciprocal etiology of this numerous class of affections. This chemico-vital pathology is not only more satisfactory in itself, but what is more important, it unfolds therapeutical indications much more definite and precise. Keeping in view the great physiological purposes of the liver and lungs, their common or vicarious relations, the intimate connection between them and the other emunctories of carbonaceous principles, the therapeutist will be able to administer the powerful class of carbonagogue remedies, with a judgment and discrimination unattainable in any other way. The hitherto vague practical generality known as "the restoration of the secretions" assumes thus an intelligible form and a practicable meaning.

It has been very certainly ascertained that the amount of carbonic acid gas exhaled by the lungs is materially influenced by the temperature of the external air; being greatly increased when the temperature is very low, and diminishing as the mercury rises. The necessity for a varying production of animal heat, and the modification of atmospheric density explain this fluctuation. The repair and waste of the tissues remaining unchanged, a hypercarbonised condition of the blood must ensue, under an elevated range of the thermometer, unless this be prevented by an increased functional activity of other organs than the lungs. In warm climates and warm seasons of the year, this increase of function mainly devolves upon the liver and the skin; increased cutaneous transpiration and hepatic secretion carry off from the system, in high temperatures, the carbonaceous materials which, under opposite circumstances, find egress through the lungs. The hepatic secretion expels these materials in an unoxidized form, and thus lessens the generation of animal heat. Increase of function engenders an increase of predisposition to disease; hence, while pulmonary diseases largely predominate in high latitudes, affections of the liver and other abdominal organs progressively augment as we descend towards the equator. The increase of physiological duty carries with it a greater proneness to pathological states; this is a law of the animal economy to which there are very few, if any, exceptions.

The diseases of the liver incident to warm climates, and growing out of this single cause, are numerous. Simple functional activity may transcend the limits of health, and pass into irritation or the initial stage of inflammation. As a consequence of this organic exaltation, bile may be formed in morbid excess, and bilious diarrhea, or jaundice from redundant secretion, may ensue. A still higher erethism of the organ may lead to a partial, or complete suspension of the function, a result which may directly arise from inordinate excitement, or indirectly, from exhaustion of power by excessive action. Then, there may be inflammation, fibrinous exudation, and suppuration. Such are a few of the acute forms of hepatic disease. As a consequence of more protracted disturbance, there may be developed the several more chronic forms of disease, as hypertrophy, induration, atrophy and other degenerations of structure. Whatever be the form of the hepatic affection, whatever the character, or the name of the organic lesion, the first, the final, and the most essential effect of each and every one is the same, an arrest, or morbid modification of the great and vital offices of the gland.

As was remarked in reference to diseased conditions of the lungs, that, however their nervous, vascular, and nutritive derangements might develop a diversity of symptoms and sympathetic phenomena, and might hence demand a corresponding diversity of therapeutic observances, yet, after all, their radical importance was to be measured by their influence upon the decarbonization of the blood, and that this influence should be a governing element in the treatment; so, in diseases of the liver, the offices of this organ in consuming the melanotic blood-globules and converting them into bile, in other words, in decarbonizing the blood, are entitled to a leading place in all therapeutic considerations. The special peculiarities of the several forms of morbid action possess really no intrinsic importance apart from their influence upon this single physiological function. It is necessary to know, and it is the purpose of pathology and pathological anatomy to disclose, the successive steps by which organic lesions affecting this function are induced; this knowledge is essential to the accurate administration of such therapeutic means as will speedily contribute to the restoration of normal structure and normal function.

Some forms of hepatic disease are remediable, a few irremediable. In the former, during the persistence of the affection, the function of the organ will necessarily be abridged, and symptoms, not only of local suffering will present themselves, but remote parts, and the system at large will feel the joint influence of vital reactions, and retained excrementitious principles. The indications of cure in such cases are of a compound kind; such as look to the relief of the local exigency, and such as have for their object the elimination through unobstructed channels, as the skin, kidneys and lungs, of the retained products of textural metamorphosis. If the case be one of simple inflammation, for example, the lancet, mercury, and other antiphlogistics may be indicated for the topical lesion; while, for the relief of the poisoned condition of the general system, cholagogues, diaphoretics, diuretics, and even expectorants, may be called into requisition for their carbonagogue powers. By this composite and comprehensive course of medication, all the demands of the case will be fully responded to, and it may often happen that individual substances of the materia medica will combine qualities and powers competent to meet both forms of indication.

In hepatic diseases, as in some others, the conservative efforts of the system often point the path to be pursued, setting up increased and vicarious action in one part, to make excretory restitution for diminished action in another. The lungs, the skin, and the kidneys often spontaneously perform this office, to a limited extent, for the liver.

When there is reason to diagnose an incurable form of hepatic disease, the treatment must necessarily be restricted to palliative remedies; and these will, for the most part, consist of means calculated to allay pain, and other kinds of nervous disturbance, and to promote a compensative amount of proper excretory action. Sedatives, and carbonagogues, both medicinal and hygienic, embrace a large part of the requisite treatment.

There are several of the acute and chronic profluvia of the intestinal canal that are intimately associated with diseased conditions of the liver, and hold towards such disease, the relation of either cause or effect. Such is the fact in reference to diarrhea, dysentery, cholera morbus, and epidemic cholera. In these diseases, with few exceptions, there is a diminished, or suspended condition of the function of the liver, and often of other organs concerned in kindred physiological duties. In some, this impaired functional energy of the carbonaceous depurants of the system is only a secondary result of the disordered states of the intestinal canal, but in a majority of instances, the functional lesion is primary, the intestinal secondary. The arrest of hepatic secretion occupying the position of the causal disease, in these several affections, it is no difficult matter to render an adequate solution of such sequent results. Conjoined with the cessation of the glandular secretion, there occurs, or exists in the character of cause, an overloaded, or congested condition of the portal veins; this congestion, in its train, superinduces a similar condition of the abdominal veins generally, which, in its turn, awakens morbid irritation in the arterial capillaries of the organs implicated in the congestion, then increased exhalation from the mucous and other surfaces, and finally, inflammation and ulceration The intimate connection between the venous circulation of the liver, and of the kidneys and lungs, leads to a serious interruption of the functions of these latter organs; whilst the well-established relations subsisting between the intestinal mucous membrane and the skin, from similarity of structure and intimate nervous and vascular sympathy, cannot fail to involve, to a serious extent, the sanity of the important offices of this tissue. Thus, besides the more obvious effects resulting from hepatic lesion, expressed by the names of diarrhea, dysentery, and cholera, "there is, at the same time, an interrupton given by it to those chemical changes of the blood, and to the expulsion from it of those noxious and excrementitious principles, which it is one of the purposes of the kidneys, liver, lungs, and skin, severally to effect." In malignant cholera, the entire suspension of the urinary excretion is a familiar fact; whilst the diminished amount of carbonic acid exhaled by the lungs is a phenomenon ascertained by experimental observation, and at once inferable from the great reduction of animal heat. The same abridgment of excretory action must exist in the milder forms of this class of diseases, though to a less appreciable extent.

In diseases thus compounded of local irritations arising from physico-vital disturbances in the portal and abdominal vessels, and of more diffused and general lesions, from the retention of poisonous principles in the blood, the indications of cure are obviously, also, of a compound character.

Retained excrementitious principles may exercise a deleterious influence, not only on the nervous system, but may serve also to maintain the profluvia with which they co-exist. It is the received opinion, that the acute dropsical effusion connected with albuminous nephritis is a result of the irritation engendered in the capillary exhalent vessels by the urea, which the kidneys, in their congested and obstructed condition, are unable to eliminate; disordered or suppressed menstruation leads to vicarious eruptions of blood; the presence of bile in the blood in cases of jaundice, or even a less amount of hepatic failure, induces purpura hemorrhagica, obstinate epistaxis, and other hemorrhages; the poison productive of typhoid fever has been conjectured to cause the characteristic ulceration of Peyer's patches in its passage from the system through these structures; the fetid emanations from decomposing animal matter are very sure to excite intestinal disturbance, as so frequently happens in the dissecting apartments of our medical schools; in the same way, diarrhea, dysentery, and cholera, though mainly dependent upon more direct and efficient causes, may be aggravated and prolonged, in the manner alluded to.

The therapeutic precepts educible from these not altogether speculative pathological considerations, are sufficiently obvious. The restoration of hepatic action by remedies endowed with specific cholagogue powers, or by other remedies appropriate to the relief of the organic lesion leading to lesion of this function; the less radical auxilliary restraint of inordinate intestinal discharges, by opiates and astringents; the prevention and arrest of the secondary vascular disturbances in the bowels and other parts, recognised under the name of inflammation, by the judicious and timely use of antiphlogistic remedies; and lastly, the development of revulsive and depurative action in the skin, lungs, and kidneys, by agents severally competent to these purposes; these are the remedial means, singly or jointly administered, according to the persistence and violence of the affection, which are indicated by the pathological conditions of such diseases, and sanctioned by the results of well-tested practical application. Therapeutics thus educed from a careful appreciation of all the elements of the diseases in question, while enforcing the necessity of a paramount attention to the first link in the chain of causation, the arrest of the secretory action of the liver and its cause, are wholly opposed to that blind and routine system of practice, hitherto so prevalent, which gave exclusive regard to this single element. These therapeutic principles recognize no such exclusivism. Whilst relying mainly upon those carbonagogue remedies which are known, from long experience, to be generally adequate to the restoration of biliary secretion, they summon to the aid of these, in cases of difficulty and danger, others that are endowed with analogous influences upon other excretory organs. This philosophical mode of medication follows up diseased action from its cause to its remote effects, pervades the whole, and failing either wholly or partially, in its radical efforts, attempts the cure through less direct, and vicarious channels.

The causes, pathology and treatment of the various idiopathic fevers are topics that have occupied the speculation, research, and experimental inquiry of the profession beyond all other topics embraced within the wide range of its investigations. Without presuming to enter this extensive field of doubt and hypothesis, a few plain and well known facts, bearing upon the doctrines of fever, and at the same time, having reference to the subject-matter of this paper, are worthy of a brief consideration. Fevers have by some been divided into two great classes, according to the supposed sources of the poisons that engender them; these are, the koino-miasmatic, and the idio-miasmatic; the morbific agent, in the former class, being supposed to be in some way connected with vegetable decomposition, in the latter, with the exhalations emanating from animal bodies. They both occur under circumstances that give very reasonable probability to such hypotheses of causation. Carburetted compounds are the most abundant products of vegetable decomposition, while it is equally true that those conditions of the atmosphere most fertile in the production of typhous, and other forms of adynamic fever, are known to be generated under circumstances, which compel the presence of a large amount of such chemical combinations. Paludal effluvia, the tainted air existing in close and ill-ventilated streets and alleys, where large quantities of vegetable matter are permitted to accumulate and decay, both of which must necessarily consist of carbon in some state of combination with oxygen and hydrogen; and human exhalations emanating from the persons of the sick and the healthy, in the confined and filthy habitations of the poor, and into the composition of which carbon cannot fail to mingle, are the several recognized sources of the febrile poisons. These poisons must produce their effects, either by a direct influence upon the nervous expansions found upon the several surfaces of external relation, or by entering the bloodvessels ramifying on these surfaces, and impregnating and vitiating the blood, and through this medium, successively developing their various phenomena. This latter opinion is the one entertained by a large number of the profession, and affords a more complete and satisfactory explanation of many of the phenomena of fever than the former. An atmosphere surcharged with a poison into the composition of which carbon largely enters may prove deleterious to the system both directly and indirectly; directly, by being absorbed, and mingling in the current of the circulation, and indirectly, by displacing from the air respired, the amount of oxygen necessary to the elimination of the effete carbon constantly present in the blood as the product of the metamorphosis of the tissues.

Whatever hypothesis be entertained as to the etiology of fever, a prominent and pervading condition attending this affection, in its various forms, is an arrest, or vitiation, of greater or less duration, and greater or less intensity, of the several most important secretory and excretory processes. This arrest or impairment is universally recognized as a potential part of the febrile combination; it constitutes a link in the chain of morbid phenomena, the severance of which generally resolves the difficulty. In what consists its importance? The physiology of these secretory and excretory processes explains, at once, the noxious influences of their pathological aberrations. They are the scavengers of the system, and when they cease their labors, poisonous and polluting accumulations must occur. Of these accumulations, the product of the natural waste of the body, and possibly of the poison of fever itself, carbon constitutes an important, if not the chief element.

The restoration of the secretions and excretions forms a leading purpose of the therapeutist in the treatment of fever. The remedies which he administers have received technical names significant of their supposed powers; they are familiarly known as expectorants, diaphoretics, diuretics, and cholagogues. The chemical therapeutist, penetrating by his analytical inquisitions into the real and final purposes of these apparently diverse excretions, is enabled to condense these classes into one more definite, and to embrace many, if not most of them, under the comprehensive title of carbonagogue agents.

This view of the purposes of the excretions does not, it is true, cover the entire range of their physiological action; far from it. The kidneys and skin are known to be mainly engaged iu secerning the exhausted nitrogenous elements of the body, but they also separate a large amount of the more poisonous element, carbon. Whilst recognizing, to the fullest extent, the deleterious influences of retained nitrogenous principles, as connected with fever, and other forms of disease, it cannot be considered that an undue prominence has been given to the results flowing from the other more hurtful principle. The most that can be alleged is, that whilst carbon has been assigned its proper place in the history of morbid causation, and its agency insisted on, with some appearance of exclusiveness, nitrogenous etiology has not been fairly appreciated, and the remedies having an influence in the modification and elimination of principles of this kind rather cursorily considered. This is to some extent true. The same may be said in regard to the inorganic or saline elements of the blood, the retention of which unquestionably plays an important part in the production of disease. The pathology and therapeutics connected with the failure of nitrogenous and saline depuration of the blood are extensive topics in themselves, and demand a separate consideration. In the practice of medicine, they cannot be sundered from carbonagogue medication, for remedies having a control over the excretion of carbonaceous elements, often, at the same time, unload the system of increased quantities of the metamorphosed proteine constituents of the body. For this reason, remedial agents can rarely be administered with the single and special purpose of effecting this or the other species of elimination, for they are all more or less compound in their action; but the theory of their modus operandi may, with advantage, be separately investigated, with the effect of more fully elucidating the part which they severally enact in this composite action.

The kidneys are the organs through which the effete nitrogenous matters of the system are principally expelled. Diuretic medicines are generally supposed to conduce to the more copious and rapid expulsion of such matters. The distinction which Dr. Golding Bird very justly makes in this class of medicines, between those which merely increase the quantity of water evacuated, and those, which while effecting a like increase, also, at the same time, separate a larger amount of the solid constituents of the urine, is one of some interest in this connection. This very marked and constant difference of effect in the different articles of the class has induced Dr. Bird to divide diuretics into the two sub-classes of renal alterants or blood-depurants, and renal hydragogues; in the former subdivision, he places the saline or alkaline diuretics, in the latter, the vegetable substances endowed with the power of stimulating the kidneys. Now, it is a curious and very pertinent fact, that all the salts of the vegetable or organic acids, in their progress through the bloodvessls to the kidneys, undergo decomposition, combine with carbonic acid, and are eliminated as carbonates, by these organs. Further than this, the observation of practitioners of medicine most familiar with the curative influences of the alkaline diuretics discloses the important therapeutic fact, that alkalis given in the form of carbonates are the least efficacious as renal alterants or blood-depurants of any of the class; thus, the carbonates of potash are much less beneficial in this capacity than the tartrate or the acetate. The plain and irresistible inference from this is, that the conversion of the latter into the former, in their transit from the stomach to the kidneys, is in some way connected with their increased excellency as blood purifiers. Their consumption of the redundant carbon of the blood explains the well-attested advantages connected with the metamorphosis, and fairly entitles this portion of the class of diuretics to be ranked as carbonagogue agents.

It has been stated that remedies very rarely evince a power exclusively limited to one excretory organ; it is much more commonly true, that more than one excretion is increased, by agents endowed with well marked energy of action. This various capability admits of several explanations, but there are none more satisfactory or ingenious than the physiological one adduced by Dr. Simon. He assumes as an unquestioned fact that the various excretions are merely eliminated by their appropriate organs and not elaborated by them; that they pre-exist in the blood as products of the decomposition and decay of the body "and have their preordained outlets, and so fast as they arise, become evolved; each, as it were, at its own pole of a galvanic battery. I know nothing better to compare this process with than the phenomena of galvanic decomposition: you see the blood distributed with uniform qualities throughout the whole area of the circulation, and you see the products of its decomposition appearing with their characteristic signs at the liver, the kidneys, the skin; just as when you plunge the wires of your battery into a trough of water, you get oxygen evolved at one pole, and hydrogen at the other, while the intermediate material remains apparently unchanged. And to apply that analogy somewhat further, I would point out this for your notice; as you are quite sure in decomposing water, that for every volume of hydrogen at one pole, there must be a corresponding half volume of oxygen at the other,—as you are quite sure that, if the gas be not evolved, it must have spent itself oxidizing the metal of that opposite wire, so with the manifestations of secretion. You cannot deal with them singly; if the essential ingredients of urine, bile, or sweat, be formed in excess, you are quite sure that certain other ingredients *complementary* to them must have been formed in excess likewise. Supposing for a moment, that the liver and the kidney were the only organs to be considered, it would be a chemical impossibility for the blood to furnish material for one of these glands, without likewise evolving, as a necessary residue of that process, the characteristic elements of the other secretion. As an obvious illustration of this, I may cite an interesting observation by Dr. Bence Jones, respecting the digestive process: when much acid was secreted by the stomach, the urine was found to be alkaline: the excess of acid in the stomach was hydro-chloric, and the free alkali in the urine was fixed alkali and not ammonia; in extreme cases, the alkalinity lasted for four hours; as the free acid was absorbed from the stomach, the urine became acid; and this reaction increased, until it affected litmus paper intensely." According to this very clear and scientific exposition of the mutual dependence of the several excretions and secretions, the development of one necessarily involves the complementary development of the others. Extending this exposition beyond physiological states of the system to conditions of disease, and the remedial means requisite to subvert them, there is no reason to doubt that the principle announced will hold equally good. Upon this hypothesis of excretory action, and the farther very reasonable hypothesis, that the presence in the blood, of matters requiring elimination is often alone sufficient to rouse the proper organs to increased effort, without the aid of extrinsic influences, it may be fairly inferred that carbonagogue medicines, besides their direct action as depuratives of the effete carbonaceous matters of the blood, will also indirectly but effectively contribute to the expulsion of useless nitrogenous elements.

In the treatment of disease generally, and particularly of acute febrile disease, the restoration of the secretions, either by medication, or by the critical efforts of the system, is usually attended by a cotemporaneous mitigation of the most threatening symptoms. The establishment of free biliary secretion is esteemed especially curative. When febrile excitement is inordinate, and animal heat is largely generated, an important chemical indication of cure is fulfilled by the elimination of the carbonaceous constituents of the system, in the unoxidized form in which they exist in bile; by promoting this free evacuation without the action of oxygen, febrile heat may be very materially controlled. Hence the great value of that variety of carbonagogue medicines known as cholagogues, in the treatment of such diseases. But, there are some forms of febrile disease, and stages or periods in all forms, occasionally, in which a leading and vital indication is, to sustain animal heat. This important purpose will certainly be frustrated if the physiological functions of the liver be unduly stimulated, and cholagogue remedies be indiscriminately administered. In cases of this nature, it would be well, were it possible, to restrain biliary action, instead of promoting it; but as we possess no remedies that are known to be anti-cholagogue in their action, the same end may probably be attained by arresting the peristaltic movements of the bowels by opiates and other sedatives, so that resorption of bile may take place from the gall-bladder, ducts, and intestinal canal, the constituents of which, combining elsewhere with oxygen, will produce the desired result. The sudden arrest of what is termed bilious diarrhea is very sure to be followed by febrile excitement, and this is the chemical rationale of the sequence. On the same principle partly, no doubt, opium forms one of the most reliable stimulants in low forms of fever, and other exhausted states of the system.

Experimental inquiry has furnished some approximation to an accurate estimate of the average amount of carbon excreted in its several forms, by the healthy adult, in each diurnal period. A brief statement of the details and sum total of the amount thus estimated has been already given. Much, however, yet remains to be ascertained, before such estimate can be deemed sufficiently perfect, to be confidently relied on, for practical purposes.

Reference has also been made to a few facts or observations connected with the increase and diminution of carbonaceous excretion, by the different emunctories of this substance, when modified in their structure and functions by disease. In this field of research, very little has been attempted, and still less has been attained. The important branch of pathological investigation having reference to the precise amount of carbon thrown out by the skin, the lungs and the liver, in the various diseases of these organs, or in the various more general diseases, is yet almost wholly unexplored, and remains to reward the labors of future observers with a harvest of most valuable information. There are intrinsic difficulties in the nature of the subject, which, perhaps, no ingenuity, or nicety of experimental manipulation can wholly overcome; yet, certainly, a closer approximation to a correct ascertainment of some of the most important modifications of this function by diseased action may be realized, than has thus far been secured. The difficulties to be surmounted in regard to the skin and liver, are greater than in regard to the lungs; they must be obviously greater in acute and severe forms of disease, than in chronic ones; in reference to the kidneys, very accurate information is readily accessible, owing to the facility with which the secretion of these glands may be obtained, in a state of purity; for this reason, this special department of pathological inquiry has already been sufficiently developed, to afford to therapeutics the most invaluable aid.

Correct chemical pathology of the kind just adverted to, is an essential and fundamental element of successful therapeutics. But, besides this, there is something equally indispensable, a precise knowledge of the influence of medicines upon carbonaceons depuration. We may discover that certain diseases are invariably characterized by an increased or diminished evolution of carbon, or by a transposition of the office from one organ to another, and yet, our efforts to correct the special aberration will be wholly impotent, without the aid of carbonagogue remedies, the powers of which have been definitely and clearly ascertained. Practical medicine demands for its successful prosecution, the possession of means of known and well ascertained powers, capable of being applied, with directness and certainty, to morbid conditions equally well ascertained. In this way only, can an approach be made to specific medication, so confidently expected by the patient, and so little attainable by the practitioner. Mere general statements about the action of medicines, such as we find in medical works, for the most part, are not sufficiently precise; their broad and indeterminate generality robs them of definite practical utility, and unfits them to fulfil the more delicate indications of disease.

Experimental inquiry of an accurate character has been prosecuted, to a limited extent, in reference to the class of agents discussed in this paper; a cursory review of some of the results of this inquiry in regard to a few medicines may not be uninteresting.

The opinion almost universally entertained as to the leading action of calomel in large and purgative doses is, that it possesses very certain powers as a cholagogue. The green color of the alvine evacuations so constantly ensuing upon the free administration of this remedy, has, among other things, given rise to this prevalent opinion. Experiments and chemical analyses conducted by a number of very impartial observers would seem, however, to throw some doubt upon the question, whether calomel does really promote the expulsion of the biliary secretion. The most competent of French authorities, Trousseau and Pidoux, think such action but little probable. Dr. Franz Simon and M. Mialhe, found, after large doses of calomel, a great quantity of bile and biliverdine. Dr. Golding Bird and Professor Schonbein, explain the green color of the dejections, not by the presence of an increased quantity of bile, but by an alteration of the hematosine. Dr. John Simon entertains opinions somewhat peculiar and original, in reference to the influence of all medicines upon the organs of excretion. He thinks that particular medicines increase the functions of particular organs, not because they have any special affinity for those organs, but for the reason, that by their catalytic action upon the blood, they evolve from this fluid a larger proportion of those materials which naturally find an exit from the system through such organs; he thinks that medicines which stimulate the excretory processes do so, by stimulating "that metamorphosis of their material which sooner or later furnishes the elements of discharge; that their best stimulants are their own characteristic excretions; that if these exist in the blood, no extraneous stimulation can be so effective as they, for exciting the organ to which they belong; that if they do not exist in the blood, no special stimulant of the organ which ought to evolve them can do more, even in its highest doses, than bring away from that organ the results of an immature excretory process, admixed with those of inflammatory excitement." With this view of the mode of all excretory development, he has reason for believing that calomel and other mercurial preparations may augment not only the hepatic, but several other secretions, by accelerating the waste of the tissues, and of the blood, and thus furnishing the materials essential to their elaboration. For the purpose of still further determining this important and mooted question of therapeutics, Dr. Michea instituted a series of accurate chemical analyses, the results of which are embodied in an interesting paper in L'Union Medicale. He analysed "1st. the spontaneous alvine dejections of healthy men; 2nd. the same substance, of a more or less green color, from men affected with gastro-intestinal inflammation; 3rd. the same resulting from various doses of calomel; 4th. evacuations produced by neutral salts and resinous purgatives." The conclusions fairly deducible from these analyses are the following; 1st. calomel acts in a special and direct manner on the liver; this salt occasions alvine evacuations of a peculiar color, due to an excess of actual bile, as shown by the action of nitric acid, which points to the presence of its coloring matter (biliverdine) by change of coloration, and of its albumen, by precipitating the latter. 2nd. This influence of calomel upon the biliary secretion is not constant. It varies according to certain conditions and circumstances. 3rd. The green evacuations produced by calomel are more frequent with men than women. 4th. These evacuations have a peculiar consistence, viz., a viscous liquidity, somewhat like oil, or white of eggs beaten together. 5th. In some affections of the intestinal canal, an excess of bile, to be detected by re-agents, may be found in the evacuations. 6th. Spontaneous alvine evacuations in healthy people are quite free from an excess of bile. 7th. Neutral salts and resinous purgatives exercise no direct or special influence on the liver. The alvine dejections which they produce, contain no excess of bile, remaining unaltered by nitric acid and heat." These experiments, fortified by others equally entitled to respect, conjoined with the unquestionable curative influences of the remedy, in morbid states of the liver, and in more general diseases in which hepatic disturbance is a prominent symptom, seem to place the powers of calomel as a carbonagogue, beyond all reasonable cavil. As such, it may be confidently relied on, proper regard being had to the modifying conditions and circumstances alluded to by Dr. Michea.

Notwithstanding the statement of Dr. Michea that resinous purgatives are not at all cholagogue, aloes enjoys this reputation with the profession generally, and with some observers who have directed their attention very carefully to its precise mode of action. Among these may be mentioned the curious researches of Dr. Wedekind, which induce the conviction that this article does not act directly upon the intestines, but is carried by the absorbents to the liver, the function of which it specially augments. He finds proof of this mode of action in the slowness of its effects, in the nature of the evacuations, which are bilious and have a special odor, and in the remarkable fact that, when taken in the form of enema, aloes does not irritate the lower bowels more than a like quantity of simple water, but, nevertheless, operates quite actively some eight or ten hours afterward, when absorption and its effect upon the liver have taken place.

A series of very accurate and elaborate experiments conducted by Dr. Boeker, an account of which is given in the second volume of his "Beitraege zur Heilkunde," and a synopsis published in the London Lancet for November 1850, having special reference to the real effect of medicines, according to the views presented in this essay, is worthy of attentive consideration. This consideration is due to the fact that the method adopted by him must lead to an increased certainty and simplicity of therapeutic principles, to a more accurate knowledge of the true and ultimate action of individual remedies, and to the prosecution of further experiment in the same line of inquiry. Dr. Boeker has, as yet, investigated in his peculiar way only a few remedies; to ascertain the action of these, he subjected himself as well as a large number of patients, to their repeated influence; a condensed account of the results elicited will fitly close what we have at present to say upon the subject of carbonagogue medicines.

The senega snake-root, in the form of decoction, is the first article in the list of those submitted to examination. The urine passed after the free use of this decoction compared with that evacuated when the medicine had not been used, indicated a slight increase in the quantity of urea, uric acid, the phosphates, volatile salts, and extractive matters; the increase of uric acid was especially marked. By resolving the urea and uric acid into their chemical elements, the precise extent to which the administration of senega increases the elimination of carbon and nitrogen by the kidneys may be ascertained.

An examination of the pulmonary exhalations disclosed the interesting fact that the amount of carbonic acid gas expired, during the use of the decoction, was strikingly augmented, while the volume and density of the exhaled air were also greater. In eight hundred examinations, Dr. Boeker found the frequency of the pulse diminished, and this always in proportion to the increase in the exhalation of the carbonic acid. After giving the details of his examination of the blood in a number of cases, by the microscope and by chemical analysis, he adds, "as the general result of his examinations, the decoction of senega-root especially influences, that is, increases the exhalation of carbonic acid gas; thereby it increases the consumption of blood-globules, and likewise the activity of the mucous membrane of the lungs. According to these results, he recommends the administration of senega in diseases which are founded on a hyper-carbonized or melanotic state of the blood." The inferences deduced from these experiments in regard to the physiological action of senega singularly harmonize with its well-established therapeutic influences, in certain morbid conditions of the system. Experience strongly attests its value in certain stages of sub-acute and chronic affections of the mucous membrane of the lungs. It is vaguely called a stimulating expectorant; its real and essential mode of action receives a more satisfactory explanation, from the accurate observations of Dr. Boeker.

Senega is also esteemed by some eminent American practitioners a very valuable emmenagogue. Admitting the hypothesis of Dr. Lane, that one of the offices of the uterus is to discharge the effete carbon of the system, and that in amenorhea this substance accumulates in undue and deleterious quantity, inducing a hyper-carbonized condition of the blood, we can readily comprehend how senega may prove serviceable as a carbonagogue, certainly increasing the elimination of carbon by the lungs, and probably establishing in the exhaling vessels of the lining membrane of the uterus an increased energy of action, analogous to that which it produces in the mucous membrane of the lungs.

The results procured by Dr. Boeker under the administration of colchicum, in the form of the tincture of the seeds, are exceedingly interesting. The solid matters of the urine, including the urea, uric acid, and the salts were diminished in amount; he could not discover any diuretic effect from the remedy. This result is opposed to the opinion generally entertained as to the action of colchicum, and is accounted for by Dr. Boeker, by the increased action of the bowels. The carbonic acid gas exhaled by the lungs was, at first, slightly increased, but decreased, in a remarkable manner, after the remedy had been exhibited for a few days. This decrease was cotemporaneous with a marked augmentation of the secretion of bile, and was considered by Dr. Boeker to be due to this cause. The quantity of melanotic blood-globules was diminished, the increased secretion of bile following, or giving rise to their destruction, in the hepatic system. "It results generally from Dr. Boeker's examination that in the beginning of its use, the tincture of colchicum increased the exhalations of the lungs and the skin, and the secretion of the liver; but, by a few days of continued use, the effect on the secretion of bile becomes more apparent, and ultimately causes a diminution of the exhalation of carbonic acid from the lungs." With this clearly defined knowledge of the leading modes of action of colchicum, its therapeutic applications as a carbonagogue remedy may be directed with more precision than heretofore.

During the administration of belladonna in the quantity of one and a half grains in the twenty-four hours, most of the solid matters of the urine were increased, except the uric acid and the non-combustible salts. This remedy is therefore promotive of the destructive assimilation of the plasma of the blood. In the earlier periods of its exhibition, an increased amount of carbonic acid gas was exhaled by the lungs; but, when continued for some days in full doses, this exhalation diminished, while the secretion of bile and alvine evacuations were simultaneously much more free. "From the analyses and microscopical observations of the blood, it became evident that the quantity of melanotic blood-globules and of solid matters of the blood decreased, a result which is quite in accordance with those elicited from the examinations of urine and exhaled air. Dr. Boeker adds, that the use of belladonna promotes the secretion of the bile, and he observed an increase in the alvine evacuations nine times out of ten cases during the administration."

The amount of the solid constituents of the urine was slightly increased, during the exhibition of the tartrate of antimony and potash. The proportion of carbonic acid gas exhaled by the lungs was greater than natural in all of the examinations, the inspirations less frequent, and the pulse slower. The blood analysed and microscopically examined displayed always a loss of melanotic blood-globules, after the use of this preparation. "As the general result of these observations, the author supposes that the potassio-tartrate of antimony first influences the blood, and thence causes many functional alterations, especially of the stomach, liver, pancreas, &c.

Sulphur was found to induce only a very small increase of the solid constituents of the urine. The exhalation of carbonic acid gas by the lungs was, however, remarkably augmented; this effect was produced when the medicine was administered in moderate doses; when given in large doses for some time, a decreased exhalation was observed, corresponding with an increased secretion of the bile. "The quantity of melanotic blood-globules diminishes in the same proportion as the exhalation of carbonic acid increases, and generally it is observed that a proportionate decrease takes place with respect to the solid substances of the blood, the serum, the albumen, the fat, the fibrin, the blood-globules and the crassamentum. These results of examinations as to the effect of sulphur agree very well with those obtained in the practice of physic and known to every practitioner."

Observations were made by Dr. Boeker upon the intimate mode of action of several other remedies, and with very analogous results; those already adduced are sufficient in number and character to serve as illustrations of the proper method of investigating the essential action of medicines, and at the same time, of carbonagogue agents.

